# One-Step Laser Encapsulation of Nano-Cracking Strain Sensors

**DOI:** 10.3390/s18082673

**Published:** 2018-08-14

**Authors:** Chan Park, Hyunsuk Jung, Hyunwoo Lee, Sunguk Hong, Hyonguk Kim, Seong J. Cho

**Affiliations:** School of Mechanical Engineering, Chungnam National University, 99 Daehak-ro, Yuseong-gu, Daejeon 34134, Korea; cksdl4608@naver.com (C.P.); gustjr33333@naver.com (H.J.); goohala9191@naver.com (H.L.); ghdwjdgkr123@naver.com (S.H.); guddnr252@naver.com (H.K.)

**Keywords:** strain sensor, waterproof and dustproof sensor, laser encapsulation, sealing

## Abstract

Development of flexible strain sensors that can be attached directly onto the skin, such as skin-mountable or wearable electronic devices, has recently attracted attention. However, such flexible sensors are generally exposed to various harsh environments, such as sweat, humidity, or dust, which cause noise and shorten the sensor lifetimes. This study reports the development of a nano-crack-based flexible sensor with mechanically, thermally, and chemically stable electrical characteristics in external environments using a novel one-step laser encapsulation (OLE) method optimized for thin films. The OLE process allows simultaneous patterning, cutting, and encapsulating of a device using laser cutting and thermoplastic polymers. The processes are simplified for economical and rapid production (one sensor in 8 s). Unlike other encapsulation methods, OLE does not degrade the performance of the sensor because the sensing layers remain unaffected. Sensors protected with OLE exhibit mechanical, thermal, and chemical stability under water-, heat-, dust-, and detergent-exposed conditions. Finally, a waterproof, flexible strain sensor is developed to detect motions around the eye, where oil and sweat are generated. OLE-based sensors can be used in several applications that are exposed to a large amount of foreign matter, such as humid or sweaty environments.

## 1. Introduction

Recently, increasing attention has been focused on the development of flexible strain sensors that can be attached directly to the skin, such as skin-mountable or wearable electronic devices for detecting human motions [1,2,3,4,5,6,7,8]. Flexible strain sensors and a variety of materials and structures, such as metallic thin films, nanoparticles, nanowires, carbon nanotubes, and sponges, have been extensively studied [9,10,11,12,13,14,15,16]. However, such flexible sensors cannot be used in real-life applications without proper encapsulation. Conventional sensors are generally exposed to various environments, such as liquids or humidity, which cause noise and shorten the lifetimes of the sensors [17]. To solve these problems, chemical; thermal; and mechanical encapsulation methods, such as liquid polymers; multi-layer sealing; resins; bar sealing; and laser sealing, have been investigated [8,10,12,13,18,19]. However, the encapsulation processes are complicated and expensive; additionally, they compromise the sensor performance [18,20].

Thermal encapsulation is a rapid and economical process and has been widely used in several fields, such as food packaging, polymerase chain reaction plate-sealer production, and glass bonding [21,22,23,24]. Among many types of thermal encapsulation, the bar-sealing process is most commonly used to bond vinyl bags and pouches [9,10,14]. In bar sealing, a Teflon-coated bar is heated to a predetermined temperature and then pressed onto a thermoplastic film to induce fusion bonding. The films are bonded by the temperature and pressure applied to the film. Using bar sealing, films can be bonded by simply applying heat. However, the method lacks versatility because heat is transferred only in a predetermined bar shape [9,10,14]. Furthermore, accurate temperature and pressure conditions are required to bond the double layers. Laser sealing is another method of thermal encapsulation that works on similar principle for bonding two glass layers. Frit is placed between the two glass layers, and a laser is focused on the frit to melt it and create the bond. This method has excellent sealing performance and produces strong local bonding. However, it is inconvenient and economically disadvantageous to use frit because it usually delaminates [19,25].

**Figure 1:**
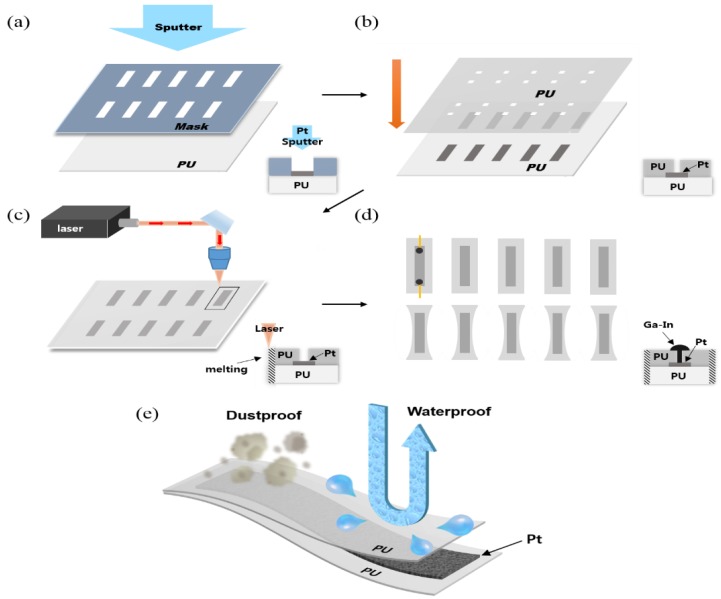
(**a**–**d**) Schematic of sensor fabrication and encapsulation; (**e**) Testing one-step laser encapsulation (OLE) sensor performance.

This study reports the development of a novel one-step laser encapsulation (OLE) method that can be applied to thin-film-based wearable devices (Figure 1). OLE is a method of bonding and cutting double layers of thermoplastic thin films using the heat of the laser. Unlike the bar-sealing process that requires pressure along with heat, OLE does not require pressure to bond thin films due to the films’ good adhesive properties [16,26]. A previously set double layer can be cut into desired shapes upon laser irradiation, and the heat provides melt-adhering, resulting in simultaneous patterning; cutting; and encapsulation in one step. An OLE sensor developed using this method has several advantages. First, three processes are simplified into a one-step process, saving cost and time. The method also enables high-speed encapsulation of sensors at the rate of one sensor per 8 s using a high-speed laser processing system (with a cutting speed of 25 mm/s). Second, the layers are tightly encapsulated with high reliability along the desired shaped edges as the laser effectively concentrates thermal energy on the focused cutting lines. Due to this tight encapsulation, the sensors have a strong resistance to humidity; high-temperature environment; chemical reactions; and pollutant exposure. In addition, the sensing layers are unharmed by the encapsulation process as the laser can be deliberately controlled to not cut near the sensing layer. Third, OLE does not require materials such as polydimethylsiloxane (PDMS), resin, or frit, which are used in other encapsulation methods, thereby making the process simple and inexpensive [11,12,13,15]. With these advantages, we expect OLE to be useful in a variety of applications, such as the mass production of thin-film-based wearable devices that can operate in sensor-unfriendly environments.

## 2. Materials and Methods

### 2.1. Materials

The materials used as sensor substrates include thermoplastic polyurethane (PU) beads (Pallethane 2363-80AE, Lubrizol, Louisville, KY, USA); Ecoflex^®^ (platinum-cured silicone rubber compounds, Ecoflex^®^ 00-30, Smooth-on, Macungie, PA, USA); Dragon Skin^TM^ (10 medium, Smooth-on, Macungie, PA, USA); polydimethylsiloxane (PDMS, silicone elastomer base, SYLGARD^®^ 184, Vision Lab Sciences, Namdong, Incheon, Korea). The PU beads were dissolved in a mixture of tetrahydrofuran and dimethylformamide (60/40; *v*/*v*) to form a 21 wt. % PU solution. Magnetron sputtering (Cressington 208HR, Oxhey, Watford, UK) was used to deposit the metallic thin film, with platinum (Pt) as the metal target. Polyester and vinylon were used as the mask material for patterning the metallic layer. Electrical connections between the sensor and the external wiring were achieved using either Ga–In (99.99+%, 495425-5G, Sigma Aldrich, Saint Louis, MI, USA) or silver epoxy (conductive epoxy, CW2400, Chemtronics, Seongnam, Gyeonggi, Korea) according to the intended application. We used polyacrylonitrile (PAN) powder (AN316020, 50 μm, Goodfellow, Spitfire Cl, Huntingdon, UK) and detergent (Cocamidopropyl betaine, Coco Bubble, Hyunjin Clean Home, Osan, Gyeonggi, Korea) to test the stability of the sensor in dusty and detergent environments, respectively.

### 2.2. Evaluation Setup

**Figure 2:**
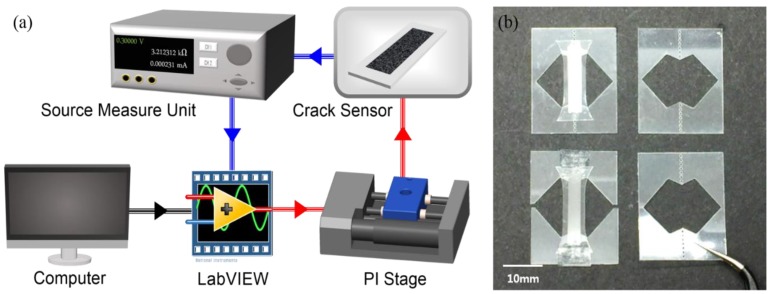
(**a**) Schematic of the sensor measurement system and (**b**) sensor handler.

Figure 2a shows the experimental setup for sensor evaluation. To obtain accurate measurements of the sensor’s electrical properties, resistance and displacement measurements were conducted using LabVIEW. The sensors were attached horizontally to minimize the effect of gravity and to control the displacement using one-axis stage. The electrical current flowing through the sensor was measured using a voltage source/measuring unit (B2902A, Keysight, Santa Rosa, CA, USA) [27]. All systems were controlled using a LabVIEW program specifically designed for this experiment. Both the controlled displacement of the stage and the resistance changes of the source/measuring unit were recorded and evaluated in real time. To address the difficulty to handle thin films, a polyester handler with dimensions of 20 × 25 mm^2^ (L × H) was designed and fabricated, as shown in Figure 2b. The handler had a hole of 1 mm in the center, which was bisected so that measurements could be performed after attaching the stage.

### 2.3. Fabrication of a Nano-Crack-Based Sensor

Herein, encapsulation was performed using a sensitive nano-crack strain sensor, which is a biomimetic analog of a spider sensor that has recently received considerable attention [27,28]. Spiders have a delicate crack-shaped organ that senses ambient vibration. As the distance between the cracks increases, the resistance also increases [28]. Mimicking such a nano-cracking structure, the sensor that was indicated in the reference was able to precisely measure the displacement and vibration with a GF of over 16,000 [28]. Because of the outstanding sensitivity and fabrication advantages, the sensor was very popular as a promising means of wearable devices despite its considerably narrow detection range (0–2% of strain). We have previously fabricated nano-crack sensors for wearable devices with a higher GF and a wider strain range as compared to those possessed by the existing stretchable strain sensors. [27,29]. The prepared PU solution was used to make a PU membrane by spin coating it onto a glass slide, controlling the thickness of the membrane via the spinning speed (thickness of ~100 μm at 250 rpm). Further, laser cut was performed at an appropriate speed (25 mm/s) to form a patterned mask. The patterned mask was placed on the PU film, and a thin layer of Pt was deposited on the membrane via magnetron sputtering for 50 s (thickness of 20 nm).

### 2.4. Thin-Film Laser Encapsulation (OLE)

Figure 1 schematizes the OLE process. After depositing the Pt layer on the PU film (Figure 1a), a covering layer was added. In the cover-layer-process step, a hole-patterned PU film (100-μm thick) was developed for wiring using a laser cutter and the two PU films were bonded via the van der Waals and electrostatic force (Figure 1b). We designed the shape of our sensor with reference to the existing tensile specimen [30,31]. The shape and the numerical value of sensors are presented in Figure S1 of the Supplementary materials. After bonding, we then used laser (25 mm/s; 4–16 W) to cut the two PU films into the desired shapes (Figure 1c). In this step, the PU films were melted locally by the laser irradiation and bonded. For this step, we used a CO_2_ laser cutter with a repeatable positional accuracy of 0.1 mm, speed of 25 mm/s, and power of 4.65 W. Next, we used Ga–In or silver epoxy to fill the hole in the upper film (Hole size: 1 mm × 1 mm (H × L)) (Figure 1d). In case of silver epoxy, it was cured at room temperature together with an electric wire for approximately 12 h. In case of Ga–In, it was applied using a syringe onto the previously fixed wires by employing a tape without any additional processing. The completed OLE sensor is protected from the external environment. Thus, the OLE sensor exhibits waterproof and dustproof characteristics, as depicted in Figure 1e.

### 2.5. Characterization Methods

The surfaces and cross sections of these devices were measured using a field-emission scanning electron microscope (FE-SEM) after the deposition of the Pt metal film. The thickness of the PU film in Figure S2 of the supplementary materials was measured using a Nikon (LV150N) microscope. We employed a Nikon AF-S NIKKOR 55–200-mm lens to obtain the general image of the sensors that was used to detect the blinking of eyes. We conducted waterproof experiments using blue inkjet ink (HP inkjet ink, CF381A, Palo Alto, CA, USA) to highlight the transparent water in Figure 4.

## 3. Results and Discussion

### 3.1. Laser Cutting and Encapsulation

To check the cutting ability of the OLE process, we tested various types of substrates and levels of laser power [15,32]. We compared the cutting conditions for three different types of substrates (PU, PDMS, and Ecoflex) at different levels of laser power relative to the conventional laser cutting process. Each substrate was prepared with a thickness of 200 μm (the sum of the top-film and bottom-film thicknesses) on a slide glass, and we performed the cutting at the power levels 4, 6, 8, 10, 12, 14, and 16 W. We classified the results of each experiment into three grades: good cutting, good cutting with burn, and poor cutting (Table 1). Details are as follows: (i) good cutting (the substrate is cleanly cut without problems such as soot, melting, or shrinkage due to heating); (ii) good cutting with burn (the substrate gets cut, but it is melted and poorly encapsulated due to shrinkage from heating); and (iii) poor cutting (the substrate is not cut). The PU film was cut at 4–6 W power without any soot. However, as the laser power level increased, the PU film was cut, but it was bent due to soot and heating. Both the PDMS and Ecoflex films exhibited similar tendencies throughout the range of laser power levels. At lower power levels, such as 4 W, neither type of substrate was cut. At 6–8 W, the PU film was accurately cut. At 10–16 W, soot and melting problems occurred; however, unlike PU, shrinkage did not occur. These results show that different substrates require different levels of laser power, which can easily be achieved by tuning the laser power output. This shows the versatility of the OLE process.

To confirm the degree of bonding between the two substrates, we analyzed the cross sections of the specimens processed with the general cutting method and with the OLE method. To evaluate the adhesion between the PU films, the adhesive force was measured by a load cell (the results of all the experiments are presented in Figure S2 of the Supplementary materials). We experimented with Figure S3a–d to ensure that the adhesive in the OLE method exhibited sufficient adhesion. One side of the square PU (10 × 20 mm (H × L)) was cut using a laser, whereas the other side was cut with a razor blade. Based on the photographs, the size of the bonded PU area was calculated (Adhesion area size: 10 mm × 150 µm = 1.5 mm^2^). The adhesion test of the same sample (Figure S3b) was performed using a load cell and stage. One side was attached to the load cell, whereas the other side was attached to the stage; further, the adhesive strength was measured by pulling the two sides (Figure S3d). The experimental results exhibit that the adhesive strength of PU on one side is approximately 2.1 N. Thus, we can confirm that the OLE method is better than other encapsulation methods and that it exhibits a force of 140 N/cm^2^ per unit area, which is 70 times higher than that exhibited by a conventional Scotch tape (3M Scotch^®^ film 720). We classified the bonding of the two substrates into two groups: homogenous bonding and heterogeneous bonding. Homogeneous bonding includes: PU–PU (6 W), PDMS–PDMS (8 W), and Ecoflex–Ecoflex (8 W). Heterogeneous bonding includes PU–PDMS (8 W), PU–Ecoflex (8 W), and PDMS–Ecoflex (8 W). We determined the power levels used for these bonding tests on the basis of previous bonding tests. For heterogeneous bonding, we set the laser power level to that of the material that requires higher power level. For example, in the case of PU (6 W) and Ecoflex (8 W), we set the power level to 8 W, based on the high output required for Ecoflex.

**Figure 3:**
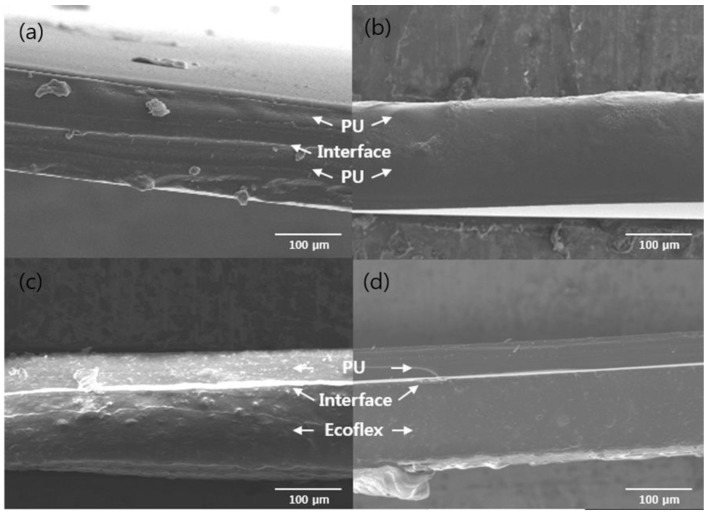
(**a**) Polyurethane-polyurethane (PU–PU) cross-sectional scanning electron microscope (SEM) image cut using the general cutting method. (**b**) PU–PU cross-sectional SEM image cut using the OLE method; (**c**) PU–Ecoflex cross-sectional SEM image cut using the general cutting method; and (**d**) PU–Ecoflex cross-sectional SEM image cut using the OLE method.

We selected one case from each bonding group (homogeneous and heterogeneous) as the representative, and the results are shown in Figure 3. Other cases showed similar results to the representative cases (the results of all the experiments are presented in Figures S3 and S4 of the Supplementary materials). Figure 3a,b show the homogeneous bonding of PU–PU substrates via razor blade and laser cutting, respectively. Figure 3c,d show the heterogeneous bonding of PU-Ecoflex substrates done by razor-blade cutting and laser cutting, respectively. In cutting with a razor-blade, the direction of the cut is uneven due to the uneven force applied during the cutting process. In addition, the thickness of each substrate is not uniform and the interfacial boundary is clearly observed between the two substrates, indicating that they are not bonded. In contrast, when using OLE, the cross sections were uniform and the interface between the two substrates could not be distinguished, indicating the formation of a single layer. However, even with OLE, heterogeneous bonding could not be achieved as the interface between the PU and Ecoflex substrates appears clearly in Figure 3d. As noted previously, general thermal encapsulation requires strong pressure for bonding layers [9,10,14]. In contrast, OLE does not require external pressure. Van der Waals force, the electrostatic force between the thin films, and the thermal energy of the laser are responsible for the bonding. For homogeneous bonding, sufficient adhesion is produced by the van der Waals force and the electrostatic force between the thin films to bond the boundary lines. However, for heterogeneous bonding, adhesion between the two substrates was insufficient without additional pressure. Therefore, both razor-blade cutting and laser cutting failed to fully bond the two substrates. Thus, the OLE method is optimized for bonding thin films of the same material.

### 3.2. Evaluation of Sensor Stability

**Figure 4:**
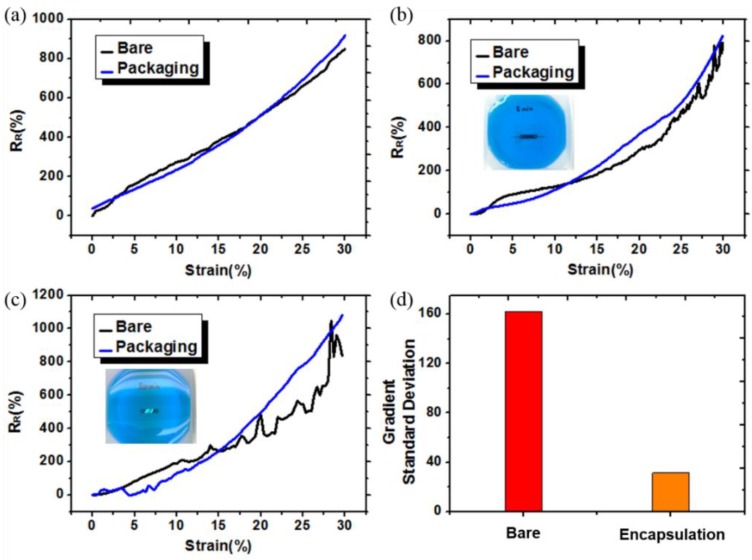
(**a**–**c**) Change in R_R_ value of a bare and an OLE sensor according to the water-exposure time (0, 5, and 50 min, respectively); and (**d**) comparison of the slope standard deviation after 50 min exposure in water.

**Figure 5:**
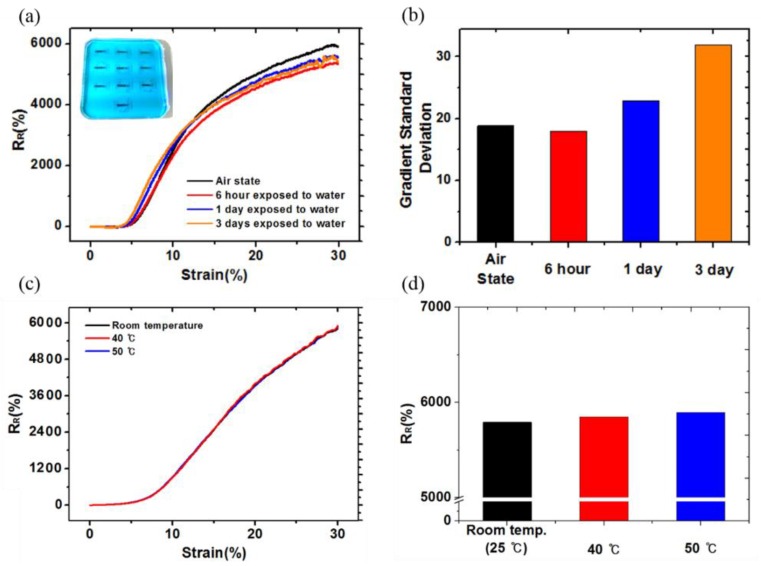
(**a**) Average relative resistance (R_R_)-strain curve of the sensors when they are exposed to water for long time; (**b**) gradient standard deviation of the sensor when it is exposed to water for long term; (**c**) the R_R_-strain curve of the sensor exposed to various temperatures (25 °C, 40 °C, 50 °C); and (**d**) R_R_ measured at 30% strain exposed to various temperatures.

The nano-crack stain sensor fabricated using the OLE process showed electrical stability in various wetting environments, such as water and sweat. To confirm the reliability of the OLE sensor in wet environments, we performed the experiments shown in Figure 4a–c. To confirm the waterproof performance of OLE, we developed an OLE strain sensor and a bare sensor and exposed them to deionized (DI) water for 0, 5, and 50 min (we dyed the DI water blue for better visualization). In the case of the bare sensor, the process of making the OLE sensor is similar, except the step depicted in Figure 1b (one layer PU). To evaluate the performance of the sensor, we measured the relative resistance R_R_ as a function of the applied strain. Here, R_R_ = (∆R/R_o_)/ε, where R, ∆R = R_on_ − R_off_, and ε denote the resistance, change in resistance, and applied strain, respectively. When the two sensors were not exposed to water, there was almost no difference in the values of R_R_ or GF [GF = (ΔR/R_off_)/ε] (see Figure 4a). However, after 5 min of exposure in water, differences between the two sensors began to appear. The OLE sensor showed a more stable signal during the entire strain test, whereas the bare sensor showed unstable resistance readings at ~20–30% of strain (Figure 4b). Even after 50 min, the OLE sensor showed a stable signal, whereas the bare sensor exhibited even greater noise. The noise level was relatively small ~10–20% strain, but severe noise was generated at 20–30% strain (Figure 4c). From numerical calculations, we found that the standard deviation of the slope for the bare sensor was eight times larger than that of the OLE sensor after 50 min in water (Figure 4d). Figure 5a is an R_R_-strain graph that exhibits the average of the ten sensors that are exposed to water conditions for up to 3 days. Additionally, the sensors were tested in long-term wet and high-temperature environments (Figure 5a). From 6 hours to 3 days, the R_R_ value decreased slightly as compared to that observed at the air state but remained similar in value. The noise was generated at 20–30% strain by exposing to water; however, it was only 1.5 times as high as the maximum at 3 days as compared to the air state (Figure 5b). We conclude that the OLE protects the sensor from water for a long time and helps the sensor to retain its electrical stability without compromising the sensing performance. Figure 5c depicts the sensor stability at room temperature (25 °C) to 50 °C. In case of an OLE sensor, it is considered that there is no need to perform a high-temperature environment test at more than 50 °C because it is a sensor that has to be attached to the human body. The R_R_ value at each temperature exhibited an almost perfect match up to 30% strain and exhibited no noise. The R_R_ value in Figure 5d was almost similar to that observed at a 30% strain.

**Figure 6:**
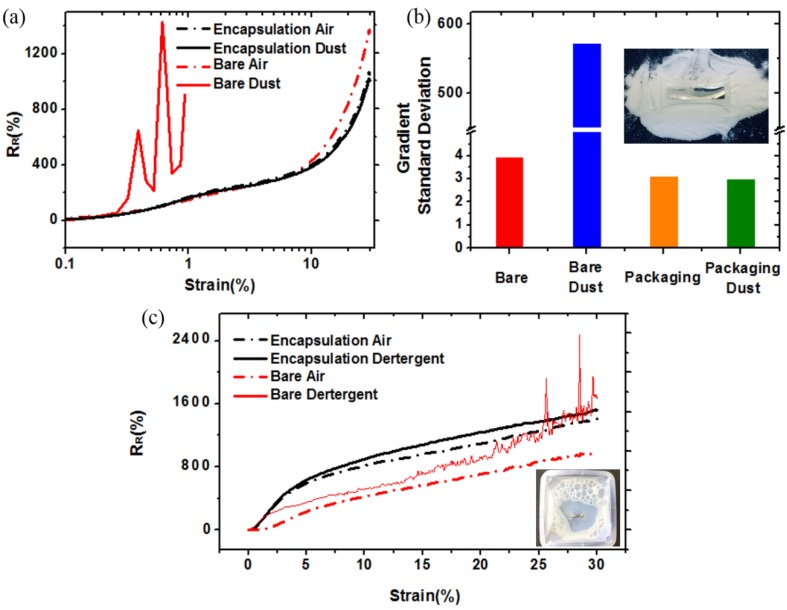
(**a**) Comparison of an OLE sensor and a bare sensor in a dusty environment; (**b**) comparison of the slope standard deviation during sensor exposure; and (**c**) comparison of an OLE sensor and a bare sensor in detergent.

To attach a sensor to the body, we require reliability in dusty and various chemical environments in addition to wet environments. We therefore compared the performance of an OLE sensor and a bare sensor during exposure to foreign matter in the surrounding environment (dust and detergent). Figure 6a shows the performance of the sensor in a dusty environment. To control parameters other than the sensing area, we used silver epoxy to wire both sides of the sensor instead of Ga–In, which was greatly affected by dust. We employed PAN powder (50 µm) as the dust material. We subjected two OLE sensors and two bare sensors to strain up to 30%, one of each being exposed to the dusty state and the other being in air. The results showed that the OLE sensor was electrically stable, regardless of the presence of dust, and that R_R_ and GF did not fall, thereby maintaining sensor performance. For the bare sensor in air, there was no noise and R_R_ was stable. However, in the dusty state, even for a strain as small as 1%, which was associated with a very small displacement, the bare sensor showed severe noise and quickly became electrically open. Figure 6b is a graph of the slope standard deviation of Figure 6a. The bare (air state), OLE (air state), and OLE (dust state) showed small slope standard deviations of 3.9, 3.1, and 2.9, respectively, whereas the bare (dust state) showed a value of 573.6, which is ~200 times more unstable.

We also conducted experiments in detergent environments using the same experimental methods (Figure 6c). We mixed the DI water with detergent at 3 wt. % and then exposed the sensors for 5 minutes. We found that all four sensors could be stretched to 30% strain, but the bare sensor exposed to the detergent was found to be unstable for strains greater than 15%. Figure 6c thus shows that the OLE can effectively protect the sensor from water as well as from various types of foreign matter, enabling it to maintain its electrical stability and sensing performance.

### 3.3. Evaluation of a Strain Sensor with OLE

**Figure 7:**
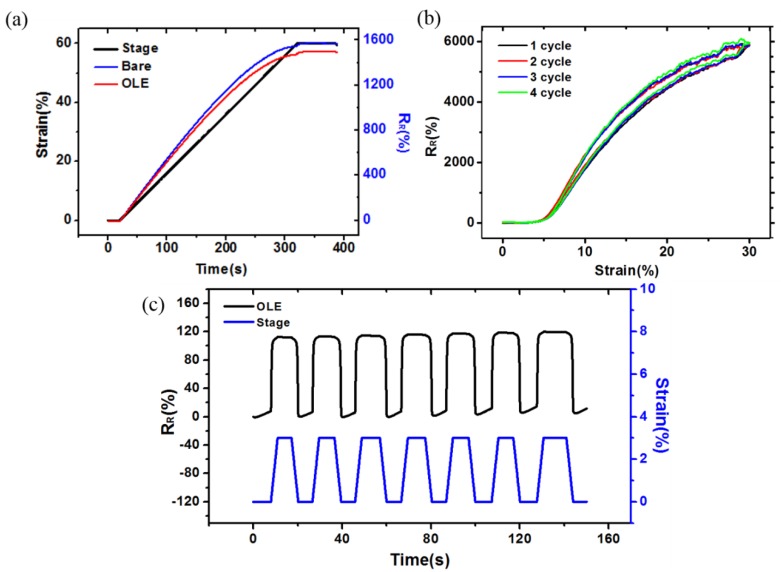
(**a**) Performance comparison between an OLE sensor and a bare sensor at 60% strain; (**b**) hysteresis at 30% strain; and (**c**) repetition experiment at low strain (0~3%).

As shown in Figure 7, the OLE strain sensor exhibited no performance degradation compared to a conventional bare sensor. Figure 7a shows the change in R_R_ of the bare sensor (blue) and the OLE sensor (red) during the tensile test for strains from 0% to 60%. As shown in Figure 7a, the R_R_ of the OLE sensor is lower than that of the bare sensor. However, it can be seen that the resistance change according to strain increases linearly with high sensitivity (GF = 33.3). Figure 7a shows the linearity and the response time, which are important performance factors when evaluating stretchable strain sensors. The graph comparing the OLE sensor to the bare sensor confirms that the OLE sensor has similar performance to the bare sensor in terms of low overshoot and fast response time (<30 ms) at 60% strain. In addition, the OLE sensor shows very low hysteresis (Figure 7b). We generated cracks through zero cycle in advance to perform accurate measurement of the sensor. Subsequently, the repetitive-strain-cycle test for strains was measured from 1 to 4 cycles. The resistance slightly increased at each cycle; however, this was a very good result as compared to that observed in case of other crack sensors [27,29]. The OLE sensor also showed excellent linearity and high sensitivity (GF = 31) even at a small displacement of 0–3% strain (Figure 7c). To conclude, we found that the OLE process does not degrade the sensor’s performance compared to a bare sensor and helps to maintain high sensitivity even in the extreme cases of low strain and large strain. With such a wide range of measurement, high sensitivity, and durability to various external environments, the OLE sensor can be easily incorporated onto human skin and excellent measurement results can be obtained. The characteristics of the OLE sensor allow direct application, even to body parts that produce exposure to several types of foreign matter.

### 3.4. Measurement of Eye Blinking with an OLE Sensor

**Figure 8:**
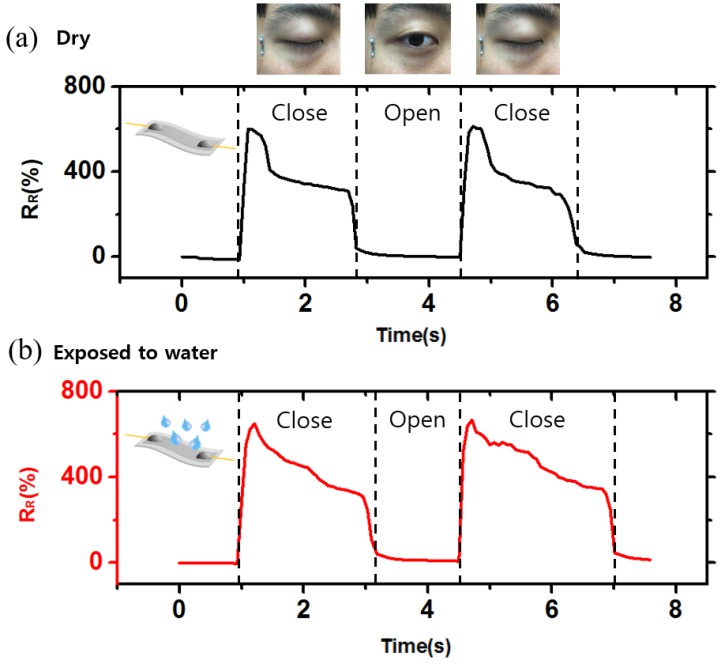
(**a**,**b**) Measurement of eyes blinking in dry conditions and when exposed to water.

To verify the practicality of the developed OLE sensor, we measured motions around the eye, where oil and sweat are generated (Figure 8a). To simulate the sweaty environment on the skin, we sprayed DI water onto the sensor. Figure 8a shows the measurement using a sensor in a dry state and attached vertically to the skin near the eye. When the eye closes, the skin is pulled and the resistance of the sensor is increased. When the eye opens again, the resistance returns to its original value. As previous results Figure 4 and Figure 5 were not related with the exposure to water, the difference between the waveforms of the two graphs was considered to be the difference between the attachment position of the sensor and the blinking behavior of the eyes. The experimental results shown in Figure 8b demonstrate that the OLE sensor performs just as well—with high sensitivity and a wide sensing range—even in environments with considerable sweat and oil. We thus expect OLE sensors to play important roles in many applications involving vigorous human activity and under various environments, such as corrosive, humid, and underwater environments.

## 4. Conclusions

This study described the successful development of the OLE method, which protects a thin-film sensor from heating and various types of foreign matter, such as water, dust, or detergent. An OLE sensor possesses several advantages. First, compared to a conventional bare sensor, an OLE sensor is protected from various types of foreign matter, thus ensuring its electrical stability, gauge factor, and wide sensing range. The OLE method, which was not applied to existing sensors, has academic value as a previously untried encapsulation method. Second, patterning, cutting, and encapsulation can proceed simultaneously in one step, which allows mass production with attendant economic merits since additional adhesive material is not required. One-step laser processing is also suitable for mass production because it can produce one sensor in 8 s. Third, an OLE sensor has stable electrical characteristics in various environments and under exposure to different types of foreign matter, and it shows good results with low hysteresis and high response speed. Considering this point, we expect the OLE method to be useful for a variety of applications, such as mass production requiring a high-temperature environment, humid environments, sweaty motions, and mounting on skin, to which it is difficult to adhere because of various types of foreign matter.

## Figures and Tables

**Table 1:** Laser cutting test using the output of each material.

Power(W)	4	6	8	10	12	14	16
PU	◎	◎	△	△	△	△	△
PDMS	X	◎	◎	△	△	△	△
Ecoflex	X	◎	◎	△	△	△	△

◎: good cutting, △: good cutting with burn, X: poor cutting.

## Supplementary Materials

The following are available online at http://www.mdpi.com/1424-8220/18/8/2673/s1, Figure S1: Sensor shape paper and PU model, Figure S2: Bonding strength by mechanical testing, Figures S3 and S4: Cross-sectional SEM image that was cut using the razor blade and the OLE method, Table S1: Cost estimation of the materials of OLE method.
