# Supplementary material for: One-Step Laser Encapsulation of Nano-Cracking Strain Sensors
Source: Sensors (Basel). 2018 Aug 14;18(8):2673. doi: 10.3390/s18082673 (PMC6111580; doi:10.3390/s18082673)
Supplement: Supplementary file 1 [file sensors-18-02673-s001.pdf]

# - Supplementary material -

One-step Laser Encapsulation of Nano-Cracking Strain Sensors

Chan Park [1], Hyunsuk Jung [1], Hyunwoo Lee [1], Sunguk Hong [1], Hyonguk Kim [1] and Seong J. Cho [1*]

[1]School of Mechanical Engineering, Chungnam National University, 99 Daehak-ro, Yuseong-gu, Daejeon, Republic of Korea; chan_cksdl4608@naver.com; hyunsuk_gustjr33333@naver.com; hyunwoo_goohala9191@naver.com; Sunguk_ghdwjdgkr123@naver.com; Hyonguk_guddnr252@naver.com

* Correspondence: scho@cnu.ac.kr; Tel.: +82-042-821-5648

Sensors **2018**, *18*, 2673; doi:10.3390/s18082673

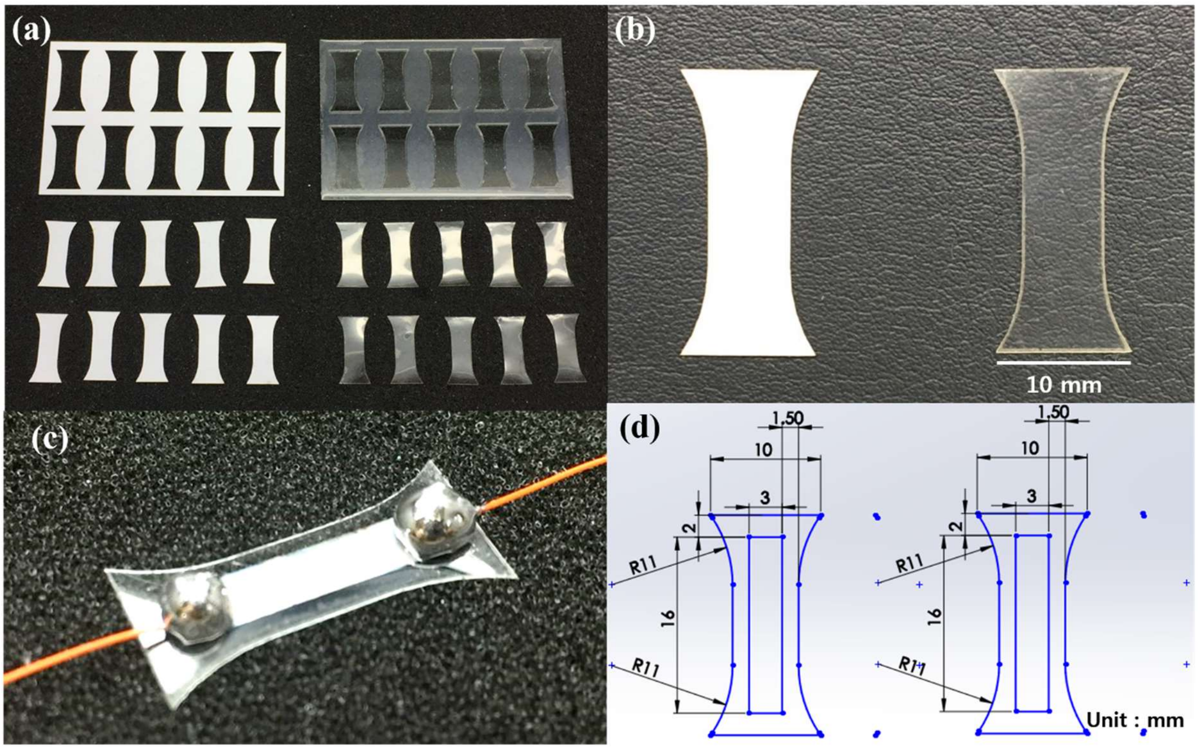

Figure S1. (a) Sensor shape paper model (left) and PU model (right), each made with laser cutting (b) Comparison of paper and PU model (c) sensor wired using Ga-In after encapsulation (d) Sensor shape design

Fig. S1 (a, b) shows that the PU is not thermally deformed by comparing the PU with the paper cut using the laser cutter. 6 W power was used for cutting. It was confirmed that there were no thermal deformation, buckling, and bending defects. Fig. S1 (c) shows an OLE sensor wired using Ga-In to demonstrate no defects such as adhesion failure, bubbles, cracks during encapsulation. Fig. S1 (d) shows the sizes of the sensor.

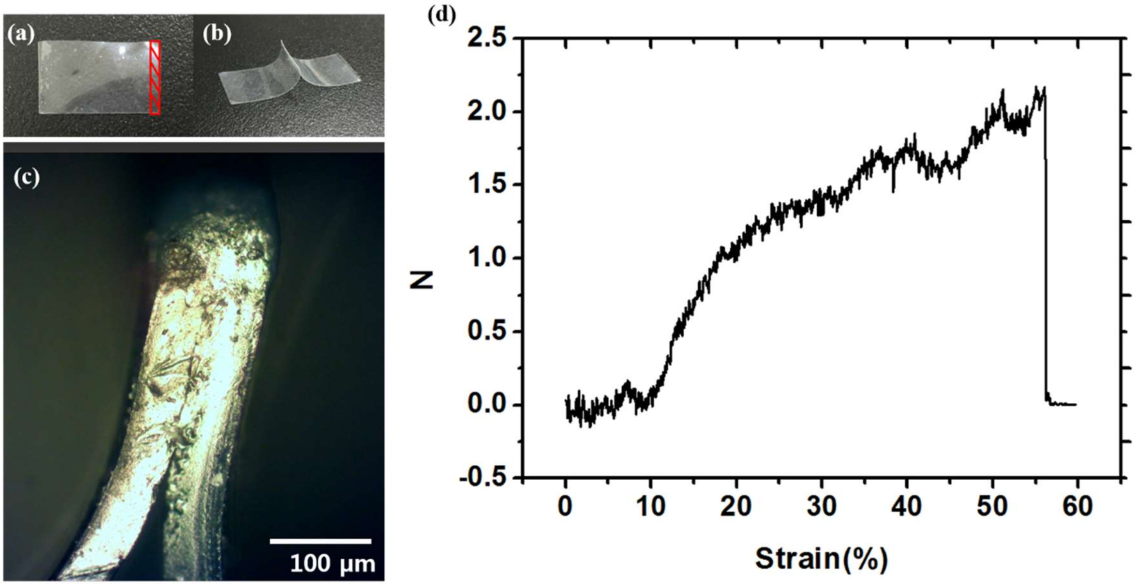

Figure S2. (a) One side of two-layers of PU was cut with a laser (marked area) and the other side was cut with a razor blade. (b) Pulling around a side bonded by laser cutting (c) Microscopic image of a side bonded by laser cutting (d) Stress-strain graph of adhesion test measured using the stage and load cell

To evaluate the bonding strength, we measured the adhesion force using a load cell as shown in the Fig. S2. Fig. S2 (a) shows the bonding area. One side of the square PU (10 mm × 20 mm (H × L)) was cut using a laser, whereas the other side was cut with a razor blade. We confirmed the bonding area and adhesive strength by pulling both sides of the PU films (Fig. S2 (b)). Based on the photographs, the size of the bonded PU area was calculated (Adhesion area size: 10 mm × 150 μm = 1.5 mm^2) (Fig. S2 (c)). The adhesion test was performed using a load cell and an automatic stage. One side was attached to the load cell, whereas the other side was attached to the stage. The experimental results exhibited that the adhesive strength of PU on one side is approximately 2.1 N (Fig. S2 (d)). Thus, we confirmed that the OLE method is better than other encapsulation methods and that it exhibited a force of 140 N/ cm^2 per unit area, which is 70 times higher than that exhibited by a conventional Scotch tape (3M Scotch® film 720).

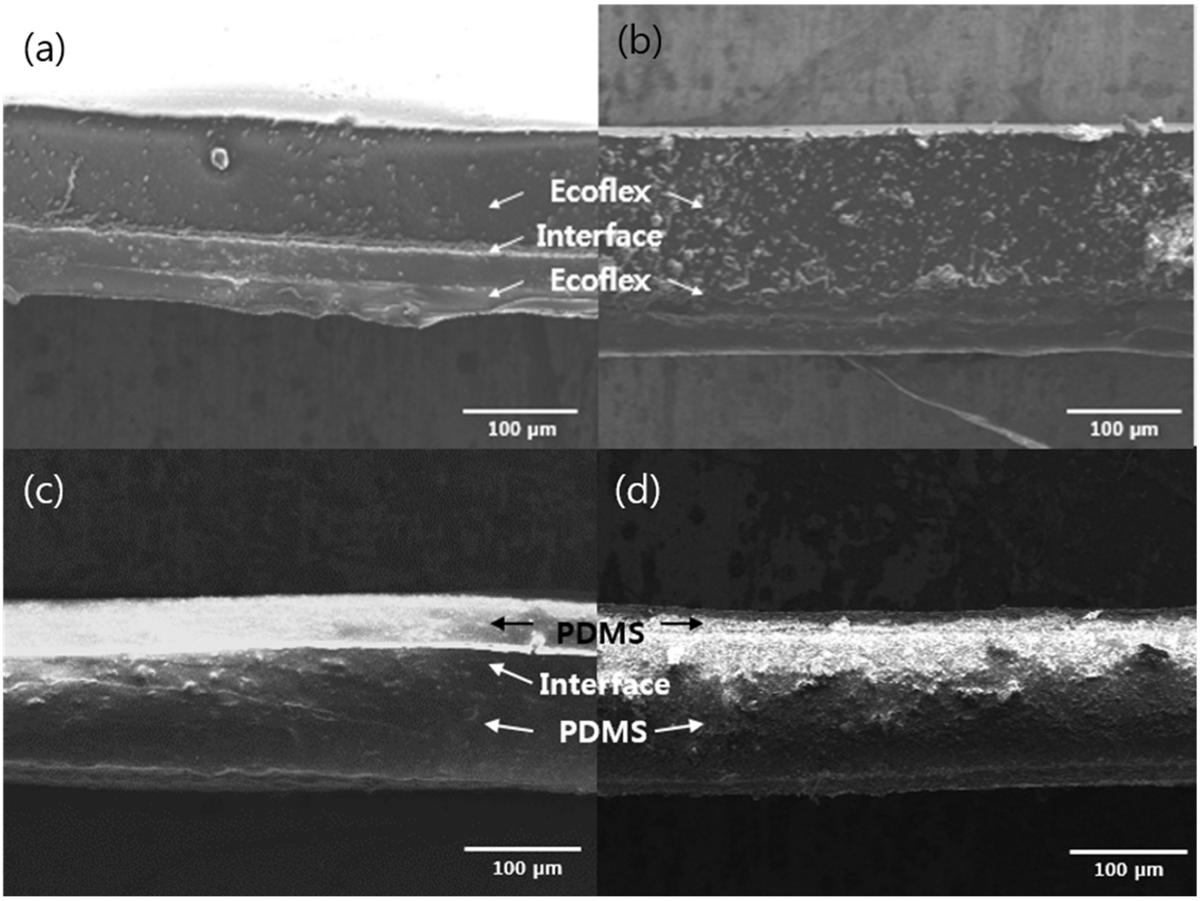

Figure S3. Cross-sectional SEM image of Ecoflex–Ecoflex cut using (a) razor blade and (b) the OLE method. (c) Cross-sectional SEM image of PDMS–PDMS cut using (c) razor blade and (d) the OLE method.

To observe the degree of bonding between the two substrates, we analyzed cross-sectional SEM images of the specimens cut with a razor blade and with the OLE method. Fig. S3 (a) and (b) show the homogeneous bonding of Ecoflex–Ecoflex substrates via razor blade cutting and laser cutting, respectively. Fig. S3 (c) and (d) show the homogeneous bonding of PDMS-PDMS substrates done by razor blade cutting and laser cutting, respectively. When cut with a razor blade, the thickness of each substrate was not uniform and the interfacial boundary was clearly observed between the two substrates, indicating that they were not bonded. In contrast, when using OLE, the thickness of each substrate at the cross section was uniform and the interface

between the two substrates could not be distinguished, indicating the formation of a single layer like that of the PU-PU substrates in Fig. 3 (b) of the main manuscript.

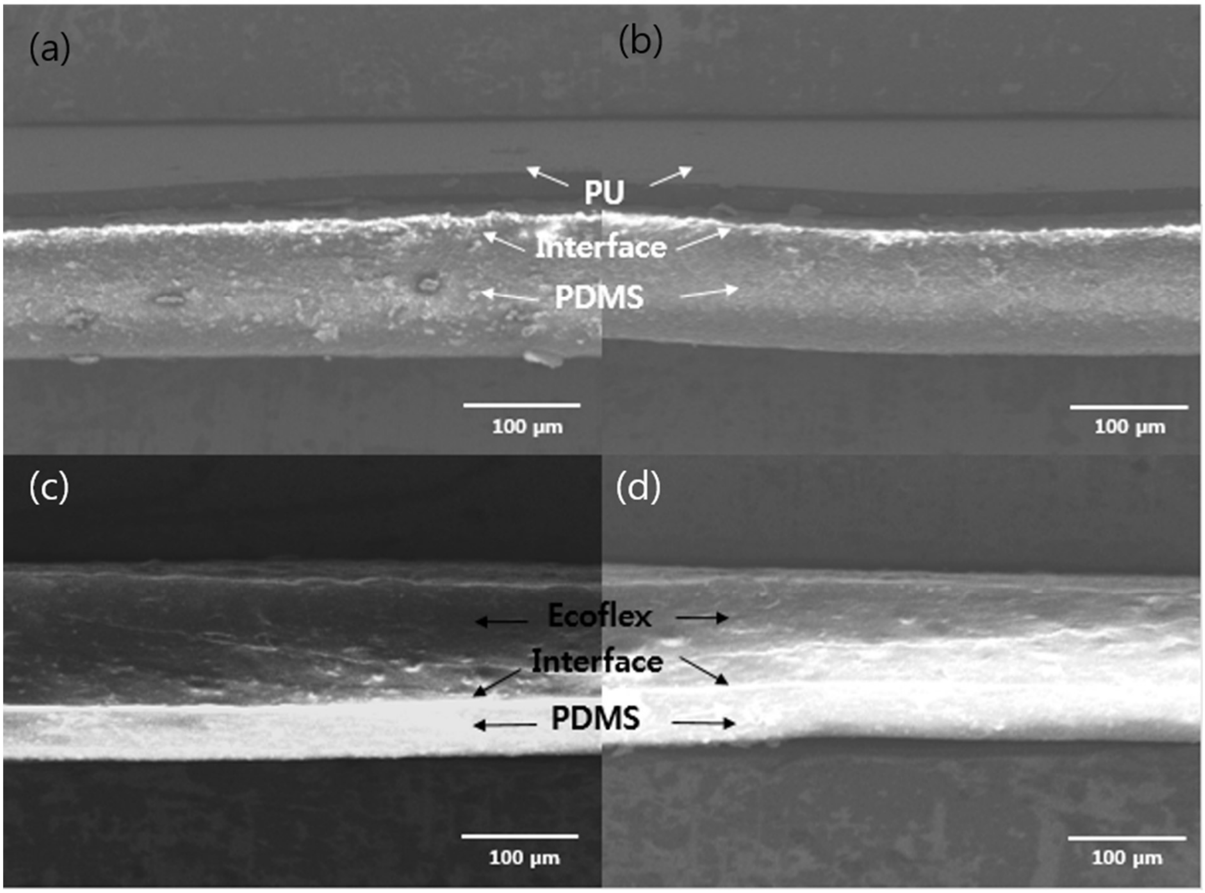

Figure S4. Cross-sectional SEM image of PU–PDMS cut using (a) razor blade and (b) the OLE method. Cross-sectional SEM image of Ecoflex–PDMS cut using (c) razor blade and (d) the OLE method.

Fig. S4 (a) and (b) show the heterogeneous bonding of PU–PDMS substrates via razor blade cutting and laser cutting, respectively. Fig. S4 (c) and (d) show the heterogeneous bonding of Ecoflex-PDMS substrates via razor blade cutting and laser cutting, respectively. Even with OLE method, heterogeneous bonding could not be achieved as the interfaces between the PU-PDMS and the Ecoflex-PDMS substrates clearly appear in Fig. S4 (b) and (d).

Table S1. Cost estimation of the materials for preparation of OLE method

Material	Commercial price ($)	Usage/device	Cost (¢ /device)
Polyurethane	$550, 5 kg / bag (Pallethane 2363-80AE, Lubrizol),	0.66456 g	11 x 0.66456= 7.31016
Tetrahydrofuran	$19, 1 L (>99.5 %, Samchun)	1.5mL	1.9 x 1.5= 2.85
Dimethylformamide	$94, 1 L (>99.0 %, Samchun),	1mL	0.94 x 1= 0.94
Platinum (Pt)	$650, Ø50 x 0.1T (>99.95 %, NRP korea),	Density : 0.0209 g / mm^3<br>Deposition volume : 48 mm^2 x 20 nm<br>Deposition weight : 2.02464 x 10^{-5} g	15700.5 x 2.02464 x 10^{-5} = 0.318
			Total cost: ~ ¢ 11.13196

Table S1 shows the estimates of OLE method materials costs. The total cost for unit device was about $0.11. We confirmed that our process our sensors are cost competitive compared to classical strain gauges ($4 ~ 5).
